# A preliminary feasibility study of a tough hydrogel as a nucleus pulposus substitute for degenerative disc disease

**DOI:** 10.3389/fbioe.2026.1821701

**Published:** 2026-08-27

**Authors:** Young-Mi Kang, Seong-Hwan Moon, Byung Ho Lee, Ji-Won Kwon, Sahyun Sung, Chang-Yk Lee, Ja-Yeong Yoon, Soo-Bin Lee

**Affiliations:** 1 Department of Orthopedic Surgery, Yonsei University College of Medicine, Seodaemun-gu, Seoul, Republic of Korea; 2 Department of Orthopedic Surgery, Ace Hospital, Ansan-si, Gyeonggi-do, Republic of Korea; 3 Yonsei Leaders Clinic, Incheon, Republic of Korea; 4 Department of Orthopedic Surgery, Chungnam National University Sejong Hospital, Sejong-si, Republic of Korea; 5 Department of Orthopedic Surgery, International St. Mary’s Hospital, Catholic Kwandong University, Incheon, Republic of Korea

**Keywords:** degenerative disc disease, nucleus pulposus, polyvinyl alcohol, silk, tough hydrogel

## Abstract

**Introduction:**

Degenerative disc disease is characterized by low back pain caused by degeneration of the intervertebral discs. Hydrogels, with favorable biocompatibility and characteristics resembling the nucleus pulposus, have received attention as potential replacement materials. In this study, we developed a tough hydrogel with enhanced mechanical performance and conducted an initial assessment of its feasibility as a nucleus pulposus substitute.

**Methods:**

The hydrogel was fabricated by incorporating 2-hydroxyethyl methacrylate–treated silk fibers into the core of a polyvinyl alcohol hydrogel. *In vitro* experiments, including cytotoxicity, cell adhesion and short-term proliferation tests, and mechanical assessments such as swelling, static compression, tensile testing, and dynamic mechanical analysis, were performed. Pilot *in vivo* studies involved implanting the hydrogel into porcine intervertebral discs, with a 21-day observation period.

**Results and Discussion:**

The *in vitro* results demonstrated no acute cytotoxicity and showed that the hydrogel supports cell adhesion and short-term proliferation, while mechanical testing indicated favorable elastic and viscoelastic properties comparable to those of the native nucleus pulposus. Histological analyses at 21 days indicated that the hydrogel remained within the nucleus pulposus space. These findings suggest that the developed hydrogel exhibits promising biocompatibility and mechanical characteristics; however, further studies are required to fully establish its suitability as a nucleus pulposus replacement for degenerative disc disease.

## 1 Introduction

Lower back pain is one of the leading causes of disability in both developed and developing countries (Hoy et al., 2010; James et al., 2018). It has been estimated that 70%–85% of all people have lower back pain at some point in their life (Andersson, 1999). In 2015, the global prevalence of activity-limiting lower back pain was 7.3% (Vos et al., 2016). In South Korea, the prevalence of the adult patients presenting with lower back pain was 8.5% in 2007 (Jhun and Park, 2009). The economic and public health burdens associated with lower back pain are substantial (Manek and MacGregor, 2005). As the number of people affected increases worldwide, the burden is expected to increase (James et al., 2018).

A well-understood pathological cause, such as vertebral fracture, malignancy, or infection, has been identified in only a small proportion of patients with lower back pain. In most cases, the cause of lower back pain still remains unclear (Hartvigsen et al., 2018; Alberola-Zorrilla et al., 2025). However, numerous studies have documented a correlation with intervertebral disc degeneration and lower back pain (Luoma et al., 2000; Cheung et al., 2009; de Schepper et al., 2010; Livshits et al., 2011; Wang et al., 2012; Teraguchi et al., 2014; Kwon et al., 2026). When degenerative changes in the intervertebral disc are confirmed to be the cause of chronic back pain, degenerative disc disease can be diagnosed.

The intervertebral disc is a fibrous cartilaginous complex that connects the upper and lower vertebral bodies and stabilizes the vertebral column. It comprises a central nucleus pulposus and an outer annulus fibrosus. The nucleus pulposus is composed of type II collagen and proteoglycans, which enable the tissue to retain high levels of water and effectively resist compressive loads (Cassidy et al., 1989; Walker and Anderson, 2004). The annulus fibrosus is a structure attached to the vertebral body and cartilage endplate. It surrounds the nucleus pulposus and resists tensile forces.

Intervertebral disc degeneration is a progressive cascade of cellular, compositional, and structural changes that is closely associated with aging (Walker and Anderson, 2004; Haefeli et al., 2006). During degeneration, the nucleus pulposus gradually loses its ability to retain water (Vergroesen et al., 2014), leading to a decrease in intradiscal pressure and a consequent reduction in disc height. The annulus fibrosus exhibits bulging, delamination, and radial fissures (Inoue and Espinoza Orías, 2011). In addition, increased microscopic and macroscopic damage to the endplate results in sclerotic changes in the subchondral bone and endplate (Rutges et al., 2011).

When severe disc degeneration is present and is accompanied by persistent lower back pain without a clear cause, the condition is generally diagnosed as degenerative disc disease. Conservative treatment approaches typically include medication, injections, physical therapy, and exercise. However, if these approaches fail to alleviate symptoms, surgical interventions such as spinal fusion may be considered (Liawrungrueang et al., 2025; Cho et al., 2026). Spinal fusion has been shown to be effective in relieving pain (Fritzell et al., 2001), but it is associated with limitations such as reduced spinal flexibility and the potential for adjacent segment degeneration (Hilibrand and Robbins, 2004; Liyis et al., 2025; McNamee et al., 2025).

The ultimate goal in the treatment of degenerative disc disease is the regeneration of the intervertebral disc and the restoration of its native mechanical function. To this end, various strategies have been explored to restore or enhance the biomechanical performance of the nucleus pulposus, including tissue engineering, cell-based therapies, intervertebral disc transplantation, and biofabrication using 3D printing. Among these approaches, nucleus pulposus replacement has long been investigated as a promising therapeutic strategy (D’Este et al., 2018); however, despite extensive research over several decades, no nucleus pulposus replacement system has yet been established as a clinically safe and effective treatment (Wilke and Sciortino, 2025). A major challenge associated with nucleus pulposus replacement is the risk of implant extrusion following implantation, and although annulus fibrosus closure techniques have also been proposed to address this issue, their clinical efficacy has not yet been conclusively demonstrated (Zengerle et al., 2020). A variety of materials, including silicone, elastic polymers, fibrin, and pyrocarbon, have been investigated for nucleus pulposus replacement applications (Wilke and Sciortino, 2025). Among these, hydrogels have emerged as particularly promising candidates for nucleus pulposus replacement.

Hydrogels have been extensively investigated as potential substitutes for the nucleus pulposus due to their high water content, viscoelastic behavior, and capacity to mimic the native extracellular matrix. Hydrogels derived from natural polymers such as alginate, hyaluronan, chitosan, collagen, and gellan gum exhibit favorable biocompatibility, biodegradability, and bioactivity (Silva-Correia et al., 2011; She et al., 2025). However, these materials generally suffer from limited mechanical strength and poor structural stability, which restrict their application in load-bearing environments (Catoira et al., 2019; Choi et al., 2019; Hu and Lo, 2021; Zhao et al., 2023).

In contrast, synthetic hydrogels such as polyethylene glycol (PEG), polyvinyl alcohol (PVA), and polyvinylpyrrolidone (PVP) offer more controllable mechanical properties, higher structural stability, and slower degradation rates (van Uden et al., 2017). For instance, PVA hydrogels fabricated via freeze–thaw cycling have demonstrated improved mechanical strength and durability, making them promising candidates for load-bearing applications. Furthermore, composite hydrogels incorporating components such as hyaluronic acid, collagen type II, and carboxymethylcellulose have been developed to combine the biological advantages of natural polymers with the mechanical robustness of synthetic systems (Reza and Nicoll, 2010; Peroglio et al., 2012; Tsaryk et al., 2015).

“Tough hydrogel” refers to a type of hydrogel engineered to overcome the inherent brittleness and low mechanical strength of conventional hydrogels, and this terminology has been adopted in numerous recent studies. Pioneering studies, such as the double-network hydrogels reported by Gong et al., demonstrated that combining a rigid, brittle primary network with a soft, stretchable secondary network can significantly enhance mechanical strength and fracture resistance (Gong et al., 2003). Subsequent work by Sun et al. introduced highly stretchable and tough hydrogels based on hybrid ionic–covalent crosslinking, further improving extensibility and toughness (Sun et al., 2012). These systems achieve superior mechanical performance through sacrificial bond breakage and effective energy dissipation, resulting in tensile strengths of approximately 1 MPa or higher, along with enhanced fatigue resistance. In the context of nucleus pulposus replacement, such mechanical properties are particularly relevant, as native nucleus pulposus tissue must withstand repetitive compressive loading and maintain hydrostatic pressure within the intervertebral disc. Therefore, tough hydrogels, which exhibit enhanced load-bearing properties and durability compared to conventional hydrogels, may serve as potential candidates for applications in nucleus pulposus regeneration and functional disc replacement. A bulk-form nucleus pulposus substitute may carry an increased risk of extrusion due to damage to the annulus fibrosus (Allen et al., 2004). To minimize annular injury, approaches involving the insertion of the substitute in the form of small-diameter fibers have been explored (Husson et al., 2003) and may be effective. However, as the diameter decreases, the material may become mechanically weaker and more difficult to handle. Therefore, it is important to maintain an optimal balance, ensuring that the fiber configuration preserves adequate compressive and tensile strength while retaining its structural form.

In this preliminary feasibility study, we developed a novel tough hydrogel matrix using PVA polymer reinforced with 2-hydroxyethyl methacrylate (2-HEMA)-treated silk fibers, and assessed its biological and mechanical compatibility via *in vitro* experiments, mechanical tests, and pilot *in vivo* implantation and evaluation in a porcine model. Through these validations, we explored the potential of using this tough hydrogel as a replacement for the intervertebral disc nucleus in the treatment of degenerative disc diseases.

## 2 Materials and methods

**FIGURE 1:**
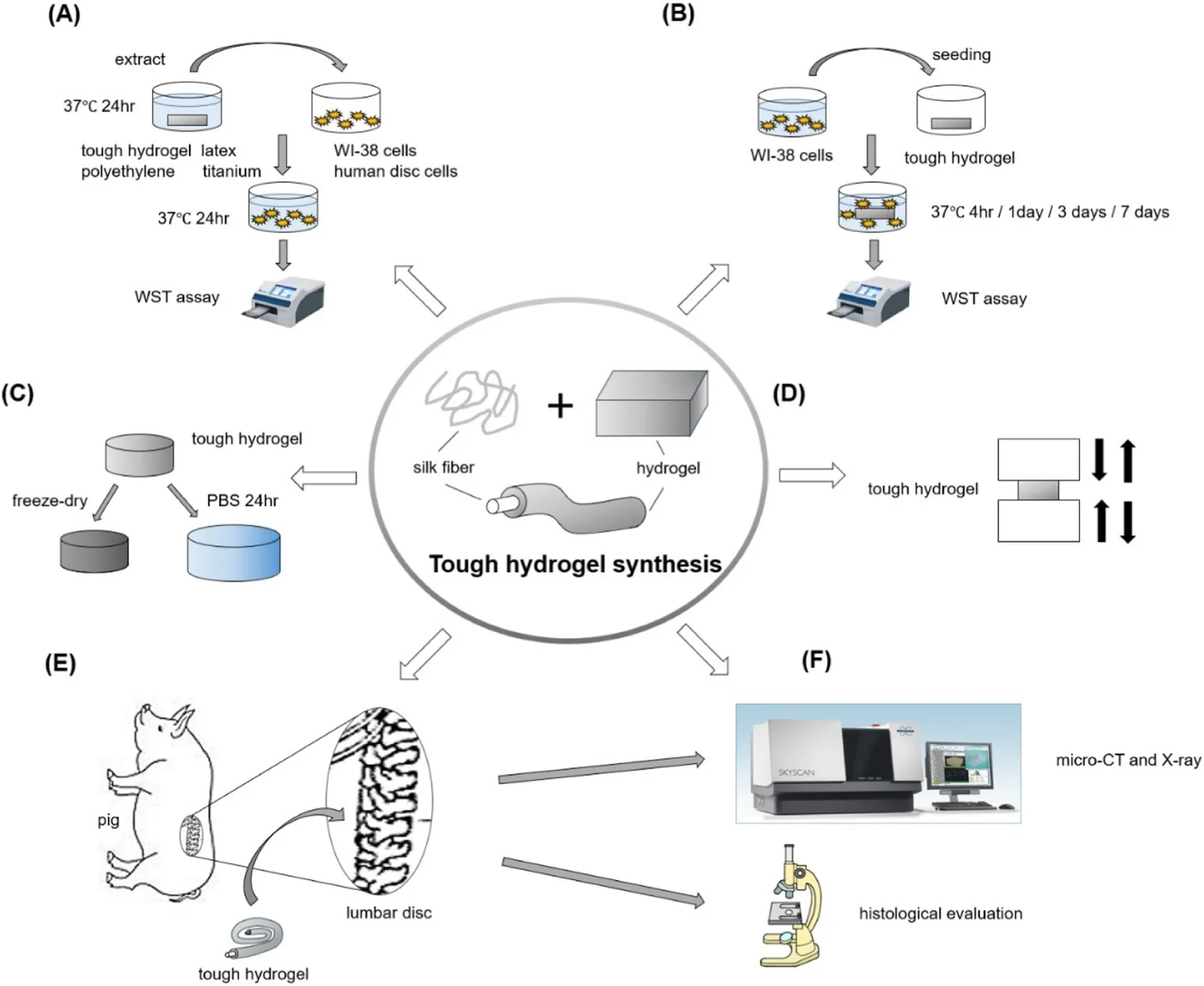
Schematic diagram of the study methods. Tough hydrogel was synthesized and evaluated in the following experiments: **(A)** Cytotoxicity test, **(B)** Cell adhesion and proliferation test, **(C)** Swelling test, **(D)** Static and dynamic mechanical test, **(E)** Pilot *in vivo* implantation, and **(F)** Pilot *in vivo* evaluation. WST, water-soluble tetrazolium salts; PBS, phosphate-buffered saline; CT, computed tomography.

A schematic diagram of the study methods is shown in Figure 1.

### 2.1 Tough hydrogel synthesis

**FIGURE 2:**
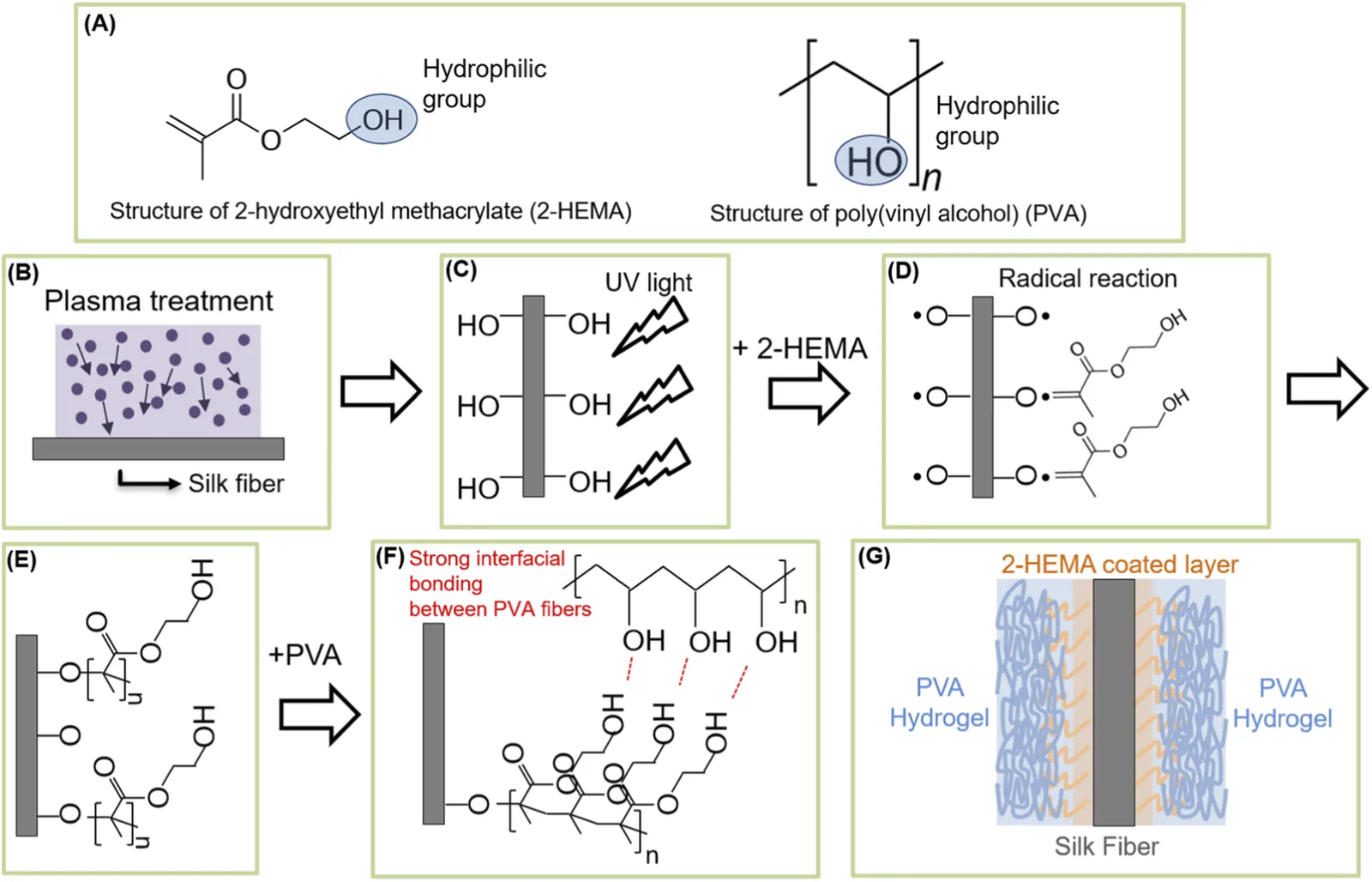
Schematic illustration of the 2-HEMA surface treatment process and tough hydrogel composite formation. **(A)** Chemical structures of 2-HEMA and PVA. **(B)** Plasma treatment of the silk fiber surface. **(C,D)** Radical reactions between hydroxyl (–OH) groups generated on the silk fiber surface and 2-HEMA, leading to covalent bonding. **(E,F)** Strong interfacial bonding formed between the hydroxyl groups of PVA and the 2-HEMA grafted onto the silk fiber. **(G)** Schematic illustration of the final tough hydrogel.

The composite hydrogel was fabricated through sequential surface modification of silk fibers followed by the freeze–thaw gelation of a PVA solution. Commercial silk fibers (KMS, Republic of Korea), approximately 35 cm in length, were used in this study. Prior to coating, the silk fibers were immersed in 70 wt.% ethanol for 1–2 h for washing and sterilization, followed by thorough washing with deionized water. The washed fibers were then vacuum-dried for 1 day before surface modification. For air-plasma treatment, the dried silk fibers were evenly spread to avoid overlapping and placed on an elevated specimen holder inside the chamber of a plasma processing system (COVANCE-1MPR, Femto Science, Republic of Korea; chamber size: 190 mm × 240 mm × 160 mm, W × D × H; maximum power: 200 W). Air-plasma treatment was performed under ambient air conditions at 100 W for 10 min to activate the fiber surface. Subsequently, the surface of the silk fiber was treated with 2-HEMA, which contains a hydrophilic group (–OH), to increase the interfacial energy between the silk fibers and hydrogel and prevent sliding. A schematic illustration of the 2-HEMA surface treatment process and the tough hydrogel composite is shown in Figure 2. One bundle of air-plasma-treated silk fibers was immersed in 100 mL of a 1 wt.% 2-HEMA solution (Sigma-Aldrich, St. Louis, MO, United States; Cat. No. 477028) containing 0.5 wt.% lithium phenyl-2,4,6-trimethylbenzoylphosphinate (LAP; Sigma-Aldrich, St. Louis, MO, United States; Cat. No. 900889) as a photoinitiator to allow surface coating of the silk fibers. The 2-HEMA-treated silk fibers were exposed to ultraviolet (UV) irradiation at 365 nm for 2 h at room temperature using a UV crosslinker (CL-1000, UVP, Upland, CA, United States; power: 8 W; maximum UV energy setting: 999,900 μJ/cm^2^) to induce photopolymerization of 2-HEMA on the silk fiber surface. Excess 2-HEMA was removed by sequential ultrasonication of the 2-HEMA-coated silk fibers in excess 70 wt.% ethanol for 12 h and excess deionized water for 12 h. The washed 2-HEMA-coated silk fibers were then dried under vacuum at room temperature for 1 day.

A 13 wt.% PVA precursor solution was prepared using poly (vinyl alcohol) (PVA; Mw 146,000–186,000, Sigma-Aldrich, St. Louis, MO, United States; Cat. No. 363065) in deionized water, autoclaved at 120 °C for 30 min, and degassed prior to use. The 2-HEMA–modified silk fiber was uniaxially aligned within a custom-designed mold and positioned at the center, into which the preheated PVA solution was injected to ensure complete embedding of the fibers. The mold was then subjected to nine freeze–thaw cycles, each consisting of freezing at −20 °C for 6 h and thawing at room temperature for 3 h, to induce physical crosslinking of the PVA matrix.

**FIGURE 3:**
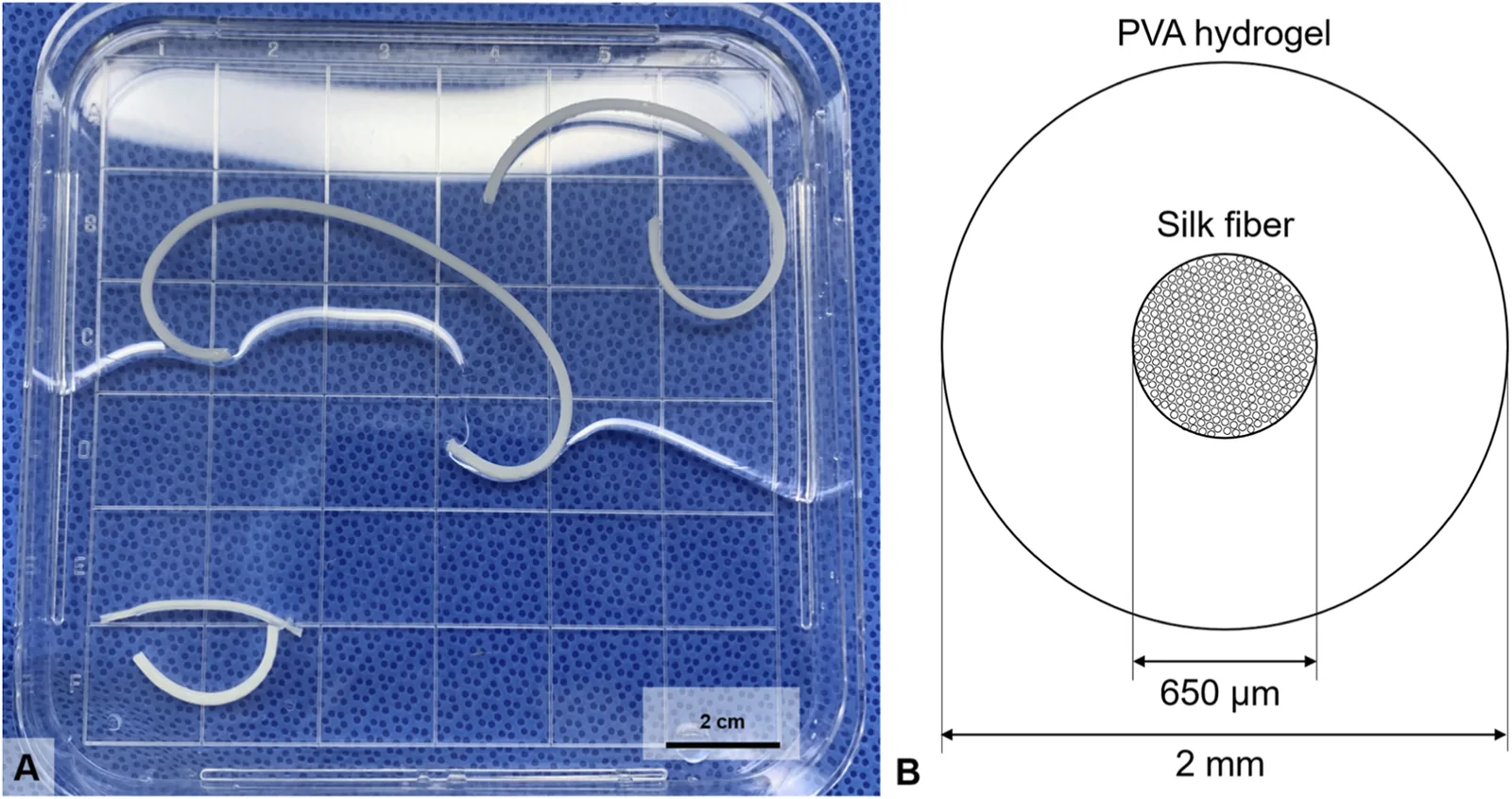
**(A)** Photograph of the tough hydrogel. **(B)** Cross-sectional schematic of the tough hydrogel. The tough hydrogel has a diameter of 2 mm and contains a silk fiber with a diameter of 650 μm in the center.

Following gelation, the construct was sterilized in 70 wt.% ethanol for 24 h and subsequently equilibrated in Dulbecco’s phosphate-buffered saline (DPBS; WELGENE, Republic of Korea; Cat. No. LB001-02) for 2 days with daily medium exchange. The resulting composite hydrogel, designated as the tough hydrogel, was stored at 4 °C until further characterization. Tough hydrogels can be fabricated in various forms; in this study, it was prepared in the form of a fiber with a diameter of 2 mm containing a core silk fiber with a diameter of 650 μm for implantation into porcine lumbar discs. Representative tough hydrogels are shown in Figure 3.

### 2.2 *In vitro* cell cytotoxicity test

*In vitro* cell cytotoxicity test was performed to evaluate the potential cytotoxic effects of the hydrogel on cell viability, ensuring its biocompatibility for nucleus pulposus replacement applications. This test was conducted in accordance with the guidelines of the International Organization for Standardization (ISO) 10993-5 (International Organization for Standardization, 2009). The tough hydrogel was sterilized and exposed to Dulbecco’s modified Eagle’s medium (DMEM; Gibco, Thermo Fisher Scientific, Waltham, MA, United States; Cat. No. 11965-092) for 24 h at 37 °C to make extract fluid. The positive and negative controls were latex and polyethylene films, respectively, and as a medically approved material, titanium was also compared. The same extraction procedure was used for each specimen. The proportion of each material and the medium was 0.1 g/mL of the medium. The fluid extract of each material and a two-fold diluted extract of the tough hydrogel were prepared. Wistar Institute-38 (WI-38) cells, which are human fibroblast-like fetal lung cells, and commercially available human nucleus pulposus cells (ScienCell Research Laboratories, Carlsbad, CA, United States; Cat. No. 4800) were seeded separately and incubated at 37 °C to a density of over 80% of the area of each culture well. The culture medium was replaced with fluid extract and the cells were incubated for an additional 24 h. After incubation, each well was washed with phosphate-buffered saline (PBS; Gibco, Thermo Fisher Scientific, Waltham, MA, United States; Cat. No. 10010-023) and incubated with the Cell Counting Kit-8 (CCK-8; Dojindo Molecular Technologies, Rockville, MD, United States; Cat. No. CK04) reagent for 1 h. Each well plate was placed in an absorbance microplate reader, and absorption was detected at 450 nm. The viability of cells exposed to each material was calculated. In addition to quantitative assessment of cell viability, live and dead cells were observed under a microscope, and cytotoxicity was evaluated using a five-grade scoring system (Supplementary Table 1). The test was performed using three independent biological replicates.

### 2.3 *In vitro* cell adhesion and proliferation test

*In vitro* cell adhesion and proliferation test was conducted to evaluate the ability of the hydrogel to support cell attachment and growth. The tough hydrogel was sterilized and prepared in a 12-well plate with a diameter of 2.1 cm and surface area of 3.9 cm^2^. A blank 12-well plate was used as a control. WI-38 cells were seeded at a density of 2 × 10^4^ cells/cm^2^ and incubated in DMEM for 4 h, 1, 3, or 7 days. At each time point, the culture medium was replaced with PBS containing CCK-8 reagent and incubated for 1 h. The absorbance of the CCK-8 reaction solution was then measured at 450 nm using a microplate reader. Cell adhesion and proliferation were evaluated by comparing the absorbance values between the tough hydrogel and the control. The test was performed using three independent biological replicates.

### 2.4 Swelling test

Swelling test was conducted to evaluate the fluid absorption capacity and equilibrium swelling behavior of the hydrogel, which are critical for mimicking the hydration properties of native nucleus pulposus tissue. A tough hydrogel specimen with a diameter of 2 mm and a length of 13 cm was prepared. The specimen was lyophilized for 24 h and weighed in its dried state. Subsequently, the specimen was immersed in PBS at 37 °C for 24 h and 7 days and then weighed in the swollen state. The test was performed using six independent specimens. The mean swelling ratio was calculated using the following equation:

$$\mathrm{Swelling ratio}\left(\%\right)=100\times \left(W_{s}\;–\;W_{d}\right)/W_{d}$$

*W_s_* = Weight of the swollen state.

*W_d_* = Weight of the dried state.

### 2.5 Static compression test

Static compression test was performed to assess the compressive mechanical properties and load-bearing capacity of the hydrogel under quasi-static conditions representative of spinal compressive loading. A cylindrical tough hydrogel specimen was immersed in PBS for 1 h and placed in Acumen® three electrodynamic test machine (MTS Systems Co, Eden Prairie, MN, United States) equipped with a 500-N load cell, and its diameter was measured. The gauge length was set to 5 mm, the specimen was compressed under displacement control at a crosshead speed of 2 mm/min using flat jigs (Supplementary Figure 1). The stiffness and elastic modulus were determined by calculating the slope of the linear region of the load–displacement and stress–strain curves. The test was performed using six independent specimens.

### 2.6 Static tension test

Static tension test was carried out to determine the tensile strength and deformation behavior of the hydrogel, which are critical for evaluating its structural integrity under tensile stresses that may arise in the intervertebral disc environment and during implant insertion procedures. A cylindrical tough hydrogel specimen was immersed in PBS for 1 h and placed in Acumen® three electrodynamic test machine equipped with a 500-N load cell, and its diameter was measured. The gauge length was set to 5 mm, and the specimen was subjected to tensile loading under displacement control at a crosshead speed of 5 mm/min using clamp-shaped jigs (Supplementary Figure 1). The elastic modulus, ultimate strength, and elongation were measured. The test was performed using six independent specimens.

### 2.7 Dynamic mechanical analysis

Dynamic mechanical analysis was performed to characterize the viscoelastic properties of the tough hydrogel under physiologically relevant loading conditions. Cylindrical tough hydrogel specimens were immersed in PBS for 1 h and placed in Acumen® three electrodynamic test machine equipped with a 500-N load cell. For dynamic mechanical analysis, a load-controlled cyclic tensile load was applied. The common elastic range of all the tested specimens, derived from the static tension test, was set as a repeated load range of 10–30 N using a sine wave. A frequency range of 1–15 Hz was set, and the analysis was performed by sequentially increasing the frequency in 1-Hz increments. The test was performed using three independent specimens. The viscoelasticity (tangent of delta, tan δ) is the ratio of the loss stiffness to the storage stiffness and was calculated using the following equation:

$$K^{*}=K^{\prime }+iK^{\prime \prime }\left(\mathrm{complex stiffness}\right)$$

δ=phase lag

$$K^{\prime }=\frac{\sigma_{0}}{\varepsilon_{0}}\cos\;\delta \;\left(storage\;stiffness\right)$$

$$K^{\prime \prime }=\frac{\sigma_{0}}{\varepsilon_{0}}\sin\delta \;\left(loss\;stiffness\right)$$

$$\tan\delta =\frac{K^{\prime \prime }}{K^{\prime }}\;\left(viscoelasticity\right)$$

### 2.8 Cyclic loading fatigue test

To simulate the repetitive loading conditions experienced in the human spine, a cyclic loading fatigue test was performed. Tough hydrogel specimens were immersed in PBS for 1 h and subsequently shaped into a spiral configuration. The specimens were mounted on an Acumen® three electrodynamic test machine equipped with a 500-N load cell. The fatigue test was performed under displacement control, and a cyclic compressive displacement corresponding to 10% strain was applied at 1 Hz for a total of 300,000 cycles, in accordance with previous studies recommending a minimum of 100,000 cycles for evaluating nucleus pulposus replacement materials (Wilke and Sciortino, 2025). The heights of the specimens were measured before and after the fatigue test to calculate the residual strain. The test was performed using three independent specimens.

### 2.9 Single fiber pull-out test

A single fiber pull-out test was performed to evaluate the effect of surface treatment on the interfacial adhesion between silk fiber bundles and the hydrogel matrix. Hydrogel specimens with dimensions of 30 mm × 20 mm were fabricated with a centrally embedded silk fiber bundle (diameter: 1 mm). Two groups were prepared: an untreated control group and a treated group in which the silk fiber bundle underwent air-plasma treatment, 2-HEMA coating, and UV-induced photopolymerization.

The pull-out test was conducted using an Acumen® three electrodynamic test machine equipped with a 500-N load cell. The hydrogel specimen was fixed while the exposed end of the fiber bundle was subjected to tensile loading under displacement control at a crosshead speed of 1 mm/min until complete pull-out occurred. Force–displacement curves were recorded during testing, and the interfacial fracture energy was determined from the area under the curve. The obtained fracture energy was used to quantify the interfacial bonding performance between the silk fiber bundle and the hydrogel matrix. Three independent specimens were tested for each group.

### 2.10 *Ex vivo* confined mechanical test

A total of six bovine caudal motion segments (Co2–Co3) were obtained from a local abattoir. All specimens were stored overnight at 4 °C in DPBS containing protease inhibitors and were equilibrated in a 37 °C saline bath for 30 min prior to testing. Each of the six biologically independent specimens underwent sequential mechanical testing under three conditions: intact, nucleotomy, and implanted. For the nucleotomy condition, a 2-mm cruciate incision was created in the annulus fibrosus, and the nucleus pulposus was removed using a 1-mm pituitary rongeur. For the implanted condition, approximately 3 cm of the tough hydrogel, prewarmed in a 37 °C saline bath for 30 min, was inserted into the nucleus pulposus cavity in a spiral-like configuration.

Following removal of the surrounding musculature and soft tissues, the motion segments were potted in poly (methyl methacrylate) and mechanically tested using an Acumen® three electrodynamic test machine equipped with a 2.5-kN load cell. Mechanical testing was performed under force control. Each specimen was subjected to a 30 N preload for 10 s, followed by 25 sinusoidal loading cycles at 0.1 Hz between −0.5 MPa compression and 0.25 MPa tension, and finally a slow ramp compression from 0 to 170 N at a loading rate of 1 N/s.

Biomechanical analyses were performed using the 25th loading cycle to ensure dynamic equilibrium. Axial range of motion (ROM), neutral zone (NZ) stiffness, compressive stiffness, tensile stiffness, NZ length, and slow-ramp compressive stiffness were calculated from the load–displacement curves. Compressive stiffness and tensile stiffness were determined by linear regression over the 60%–100% region of the maximum compressive load and the 80%–100% region of the maximum tensile load, respectively. NZ stiffness was defined as the minimum slope of the load–displacement curve within the neutral zone, and NZ length was calculated as the displacement between the intersections of the compressive and tensile linear regression lines with the neutral zone curve. Slow-ramp compressive stiffness was obtained by linear regression of the slow-ramp compression load–displacement curve (Varma et al., 2018). After mechanical testing under each condition, radiographic images were acquired, and intervertebral disc height was determined by measuring the distance between the superior and inferior endplates.

### 2.11 Pilot *in vivo* implantation in porcine model

Pilot *in vivo* implantation was performed to assess early biological responses to the hydrogel—including tissue interaction, inflammation, infection, and positional stability—and to explore an implantation approach that may be suitable for future clinical evaluation. With approval from the Institutional Animal Care and Use Committee of the Yonsei University Health System (IACUC Approval No. 2022-0224), we used three five-month-old female crossbred pigs weighing approximately 50 kg for pilot *in vivo* implantation of the tough hydrogel. The pigs were anesthetized via an intramuscular injection of alfaxan, intubated, and maintained on an isoflurane–oxygen mixture during the surgical procedure. The pig was placed in a semi-lateral position with its left side up. Draping was performed aseptically in an orthopedic manner. The intervertebral discs between lumbar vertebrae 3, 4, and 5 were accessed using an anterior approach, similar to the anterior lumbar approach commonly used in human surgeries. Level confirmation was performed using C-arm fluoroscopy. An oblique skin incision was made on the lateral side of the abdomen. Subcutaneous fat was incised, abdominal muscles were divided, and the plane between the retroperitoneal fat and psoas fascia was dissected. The peritoneal cavity was retracted medially and the psoas muscle was retracted laterally. Vertebral bodies and intervertebral discs were also identified. Under loupe magnification, an approximately 3-mm square incision was made on the lateral aspect of the disc using a No. 11 blade, and the annulus fibrosus was carefully removed. Subsequently, the nucleus pulposus was extracted using a 1-mm pituitary rongeur, taking care to avoid damage to the cartilage endplates. After nucleus removal, the fiber-shaped tough hydrogel was gently and gradually inserted into the center of the disc space using Debakey forceps, allowing it to form an internal spiral shape. The tough hydrogel was inserted into the disc space until it completely filled the cavity, being gently but firmly pushed in to a depth of approximately 3 cm. The tough hydrogel was trimmed at the annular entrance using Metzenbaum scissors, and the cut end was tucked inward to prevent it from protruding through the annular defect. Three pigs were used in the experiment, and the lumbar (L)3–L4 and L4–L5 intervertebral discs of each animal were utilized, resulting in a total of six discs used for implantation. For one pig, we implanted a 0.2-mm thick titanium fiber embedded within the tough hydrogel material to assess the positioning of the hydrogel within the disc. The surgical wound was closed layer-by-layer.

Postoperatively, the pigs were managed by veterinary staff and administered intravenous ketorolac (1 mg/kg) and subcutaneous meloxicam (0.2 mg/kg) for pain control. For antimicrobial prophylaxis, intramuscular cefazolin (30 mg/kg) was administered preoperatively, and oral amoxicillin clavulanate (0.14 mg/kg) was administered postoperatively for 7 days. The pigs were individually housed in cages measuring approximately 1.5 m × 1.5 m, with no restrictions on their activity.

### 2.12 Pilot *in vivo* evaluation

On postoperative day 21, the pigs were euthanized via an overdose of intravenous potassium chloride under general anesthesia induced by an intramuscular injection of alfaxan (1 mg/kg), xylazine (2 mg/kg), and azaperone (2 mg/kg). The lumbar 3–4–5 spine segment was harvested and fixed in neutral-buffered formalin. The sample was decalcified and sectioned at 10 μm. The sections were stained with hematoxylin and eosin and Masson’s trichrome. Disc degeneration was assessed using the scoring system described by Masuda et al. (Masuda et al., 2005) (Supplementary Table 2). In this system, the minimum possible score is 4, representing a normal disc, while the maximum score is 12, indicating severe degeneration. Segments with titanium fibers were subjected to X-ray and micro-computed tomography (micro-CT) to visualize the distribution of the hydrogel within the intervertebral disc. Micro-CT imaging was performed using a SkyScan 1173 system (Bruker, Belgium), with a reconstructed isotropic voxel size of approximately 35 μm. Images were acquired at 130 kV and 60 μA and reconstructed using NRecon with standard beam-hardening and ring-artifact corrections.

### 2.13 Statistical analyses

All statistical analyses were performed using R software (version 4.3.0, R Foundation for Statistical Computing, Vienna, Austria). Data are presented as the mean, and individual data points are shown in all figures. Welch’s *t*-test was used for comparisons between two independent groups. Repeated-measures one-way analysis of variance (ANOVA) was used for repeated measurements within the same specimens, followed by *post hoc* pairwise comparisons using Holm’s correction. One-way ANOVA was used for comparisons involving more than two independent groups, followed by *post hoc* pairwise comparisons using Holm’s correction. The statistical approach was reviewed in consultation with an independent statistician. A *p*-value <0.05 was considered statistically significant.

## 3 Results

### 3.1 Cell cytotoxicity test

**FIGURE 4:**
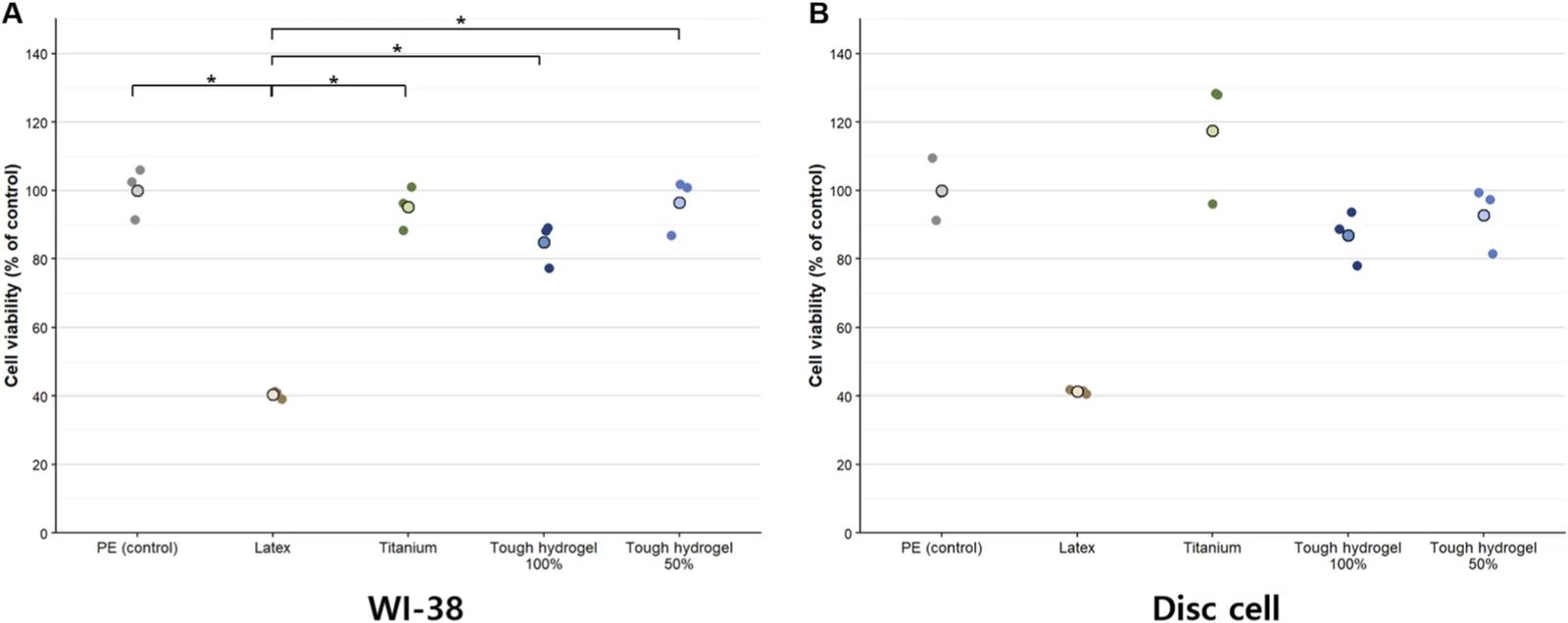
Cell viability of **(A)** WI-38 and **(B)** disc cells exposed to each material extract. Individual data points from independent experiments are shown as raw values, and group means are indicated as outlined circles. Each experiment was performed in three independent biological replicates. *Statistically significant difference with P < 0.05. PE, polyethylene; WI, Wistar Institute.

The mean cell viabilities of WI-38 cells were 84.82% and 96.49% for the 100% and 50% tough hydrogel fluid extract, respectively, exceeding the 70% threshold defined in ISO 10993-5. For disc cells, the mean cell viabilities were 86.82% and 92.83% for the 100% and 50% tough hydrogel fluid extracts, respectively. The WI-38 cell viabilities of both the 100% and 50% tough hydrogel extracts were significantly higher than that of latex, the positive control (Figure 4). In the qualitative assessment of cytotoxicity, the tough hydrogel extracts were classified as grade 0 or 1 (Supplementary Figure 2).

### 3.2 Cell adhesion and proliferation test

After a 4-h incubation period, the mean optical density of the tough hydrogel group at 450 nm was 0.21, which was 205% higher than that of the control group. The difference in optical density between the tough hydrogel and control groups increased over time (Supplementary Figure 3).

### 3.3 Swelling test

**FIGURE 5:**
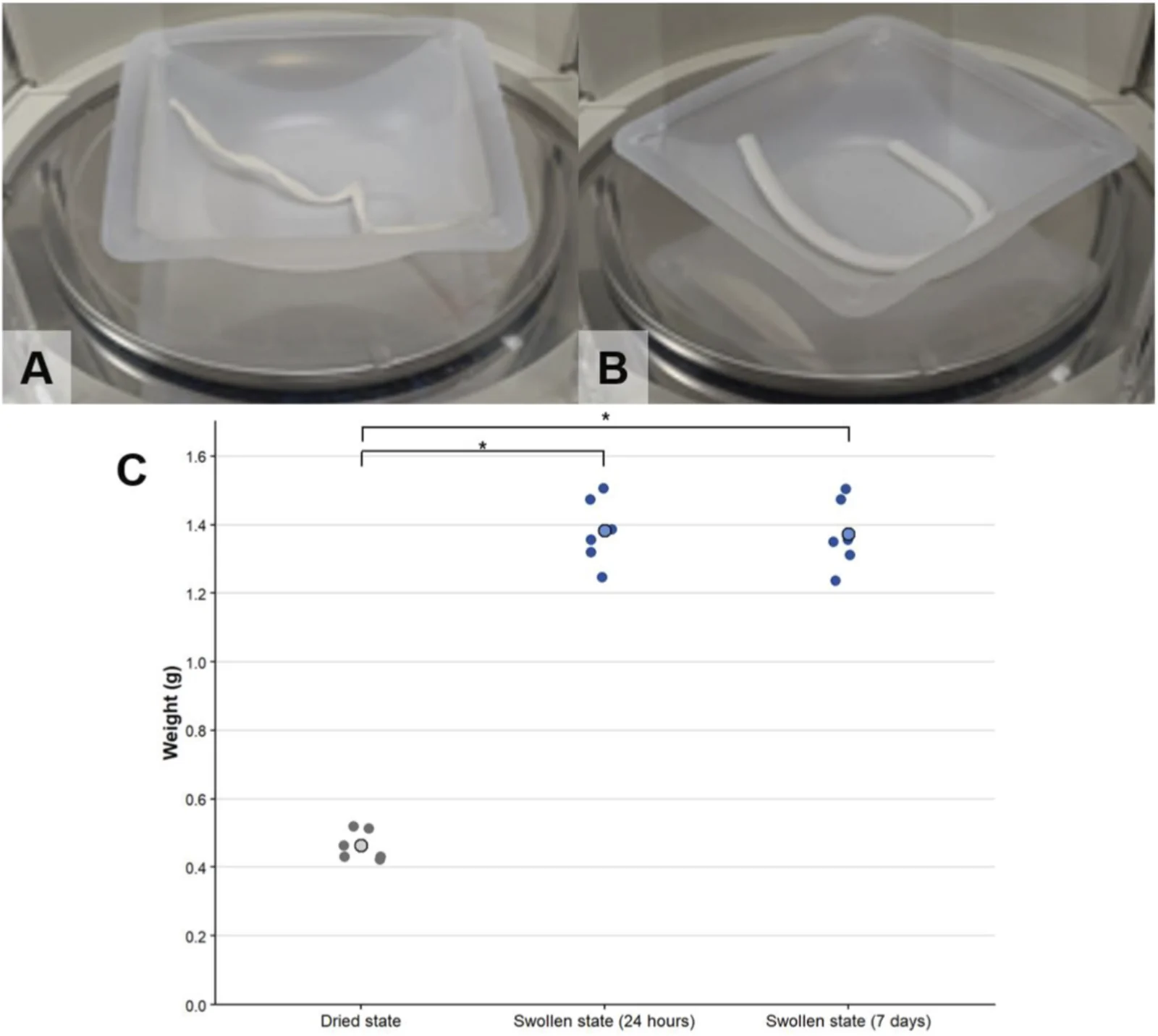
Tough hydrogel in **(A)** dried state and **(B)** swollen state. **(C)** Weight of the tough hydrogel in the dried state, 24-h swollen state, and 7-day swollen state. Individual data points from independent experiments are shown as raw values, and group means are indicated as outlined circles. Experiments were performed in six independent biological replicates. *Statistically significant difference with *P* < 0.05.

The mean weight of the tough hydrogel in the 24-h swollen state was 1.38 g, and in the 7-day swollen state was 1.36 g. Both values were significantly higher than the dried weight of 0.46 g (Figure 5). The swelling of the tough hydrogel remained constant after 24 h of immersion. The mean swelling ratio of the tough hydrogel was 202%.

### 3.4 Static compression test

The mean stiffness of the tough hydrogel specimen was 1.46 N/mm, and the mean elastic modulus was 3.75 kPa.

### 3.5 Static tension test

The mean elastic modulus of the tough hydrogel was 16.50 MPa. The mean ultimate strength was 12.54 MPa, and the mean elongation was 103.07%.

### 3.6 Dynamic mechanical analysis

The measured complex stiffness ranged from 106 to 136 N/mm and gradually increased as the frequency increased. The storage stiffness curve was similar to the complex stiffness curve. The loss stiffness exhibited a gradual decrease and a plateau pattern. The tan δ of the hydrogel ranged from 0.09 to 0.15. As the frequency increased, the tan δ decreased and reached a plateau around 0.09 (Supplementary Figure 4).

### 3.7 Cyclic loading fatigue test

After 300,000 cycles of 10% strain-controlled cyclic loading fatigue test, the tough hydrogel exhibited a mean residual strain of 7.37%.

### 3.8 Single fiber pull-out test

**FIGURE 6:**
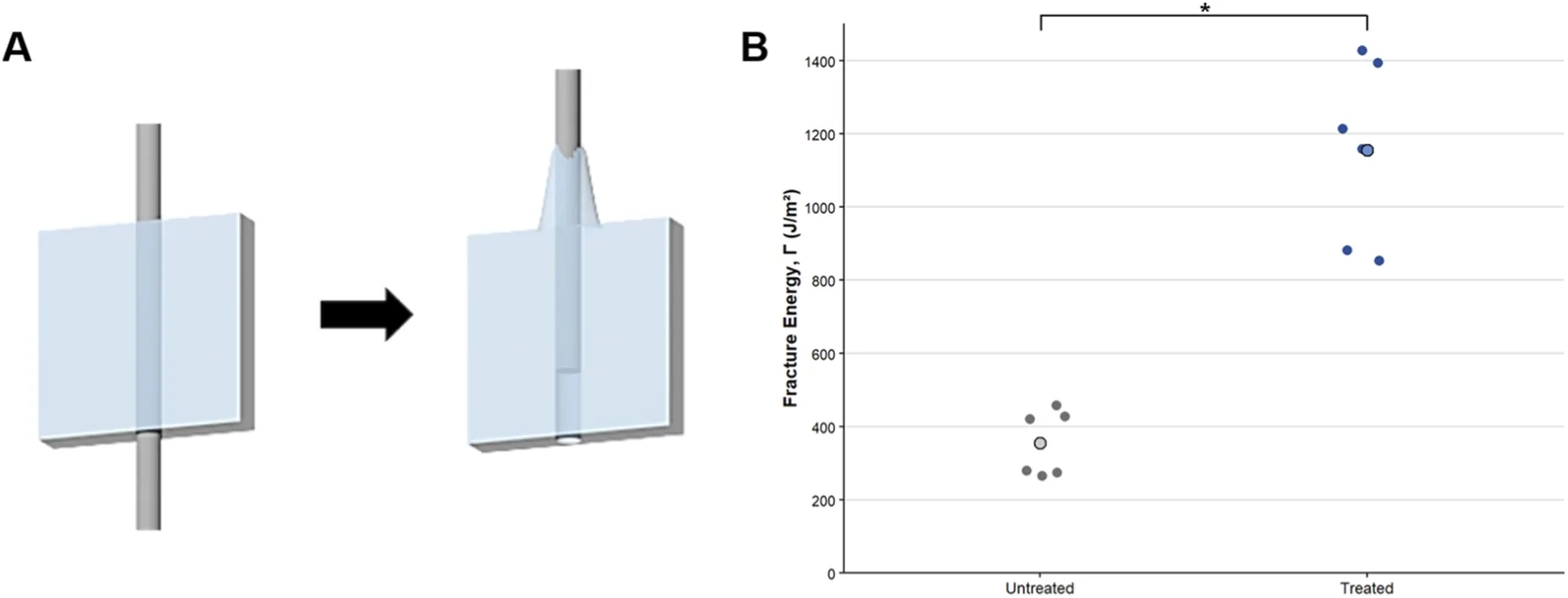
**(A)** Schematic illustration of the single fiber pull-out test. **(B)** Interfacial fracture energy of the hydrogel with embedded silk fibers, comparing untreated and treated conditions. The treated specimens were prepared using air-plasma treatment, 2-HEMA coating, and UV-induced photopolymerization. Individual data points from independent experiments are shown as raw values, and group means are indicated as outlined circles. Experiments were performed in six independent biological replicates. *Statistically significant difference with *P* < 0.05.

The surface-treated group exhibited a higher mean interfacial fracture energy of 1155.4 J/m^2^ compared with 354.8 J/m^2^ in the untreated control group (Figure 6).

### 3.9 *Ex vivo* confined mechanical test

**FIGURE 7:**
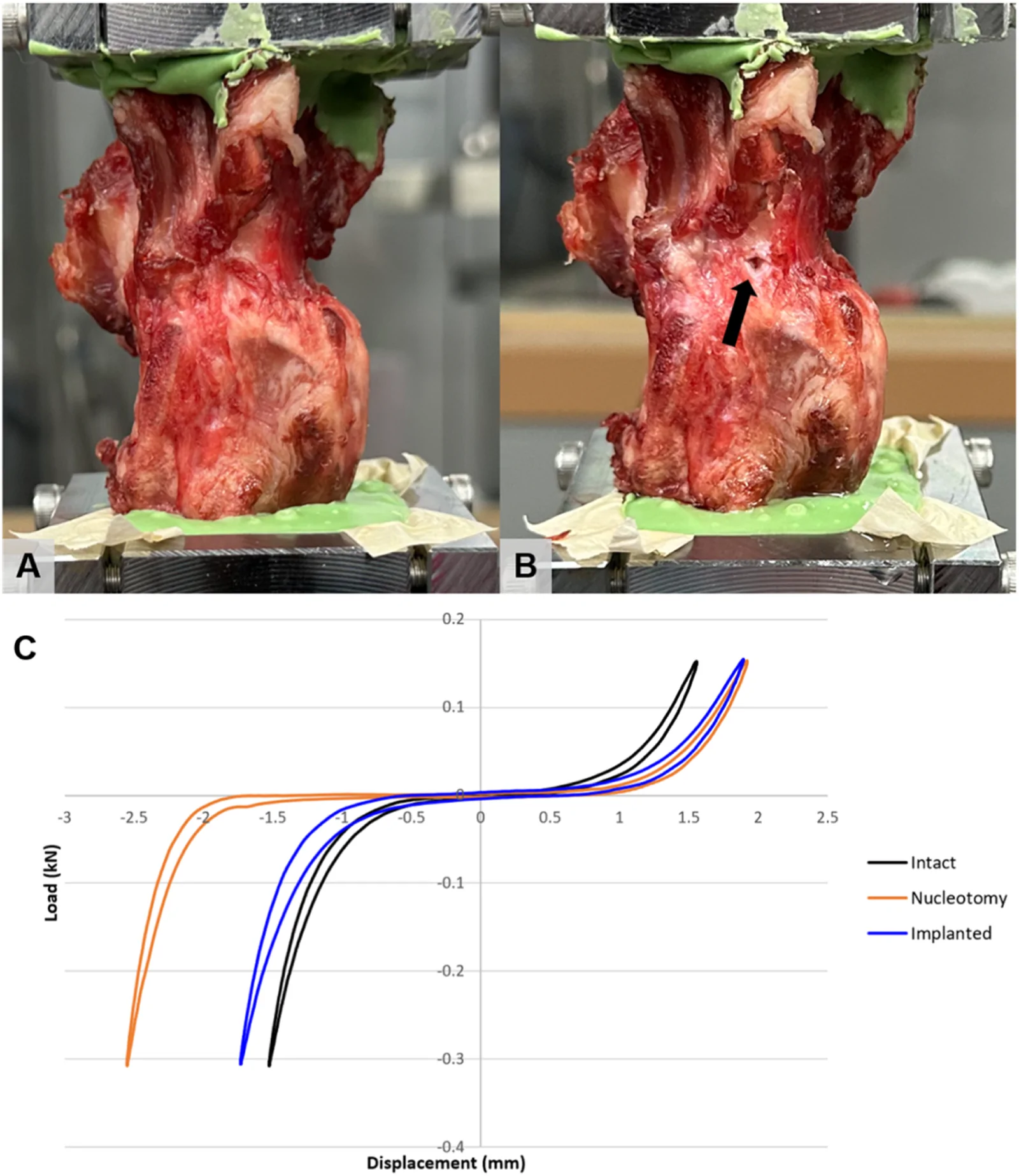
Representative images of *ex vivo* confined mechanical testing using **(A)** intact and **(B)** nucleotomy bovine tail motion segments. The black arrow indicates the annulus fibrosus incision site. **(C)** Representative load–displacement curves of intact, nucleotomy, and implanted discs.

**FIGURE 8:**
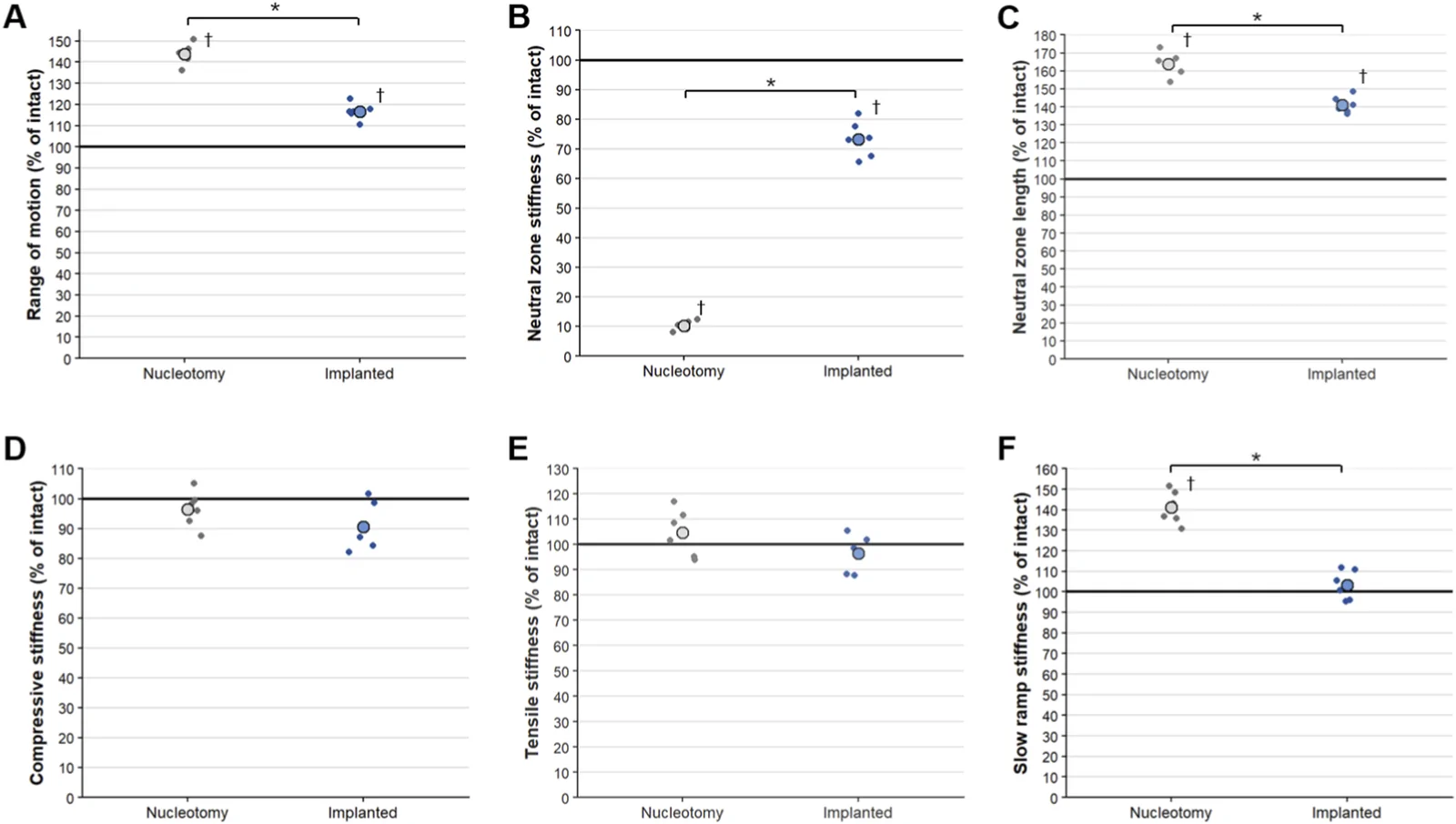
Percentage changes in *ex vivo* confined mechanical test parameters relative to the intact condition for nucleotomy and implanted discs. **(A)** Range of motion, **(B)** neutral zone stiffness, **(C)** neutral zone length, **(D)** compressive stiffness, **(E)** tensile stiffness, and **(F)** slow ramp stiffness. Individual data points from independent experiments are shown as raw values, and group means are indicated as outlined circles. Experiments were performed in six independent biological replicates. *Statistically significant difference (*P* < 0.05). †Statistically significant difference compared with the intact condition (*P* < 0.05).

**FIGURE 9:**
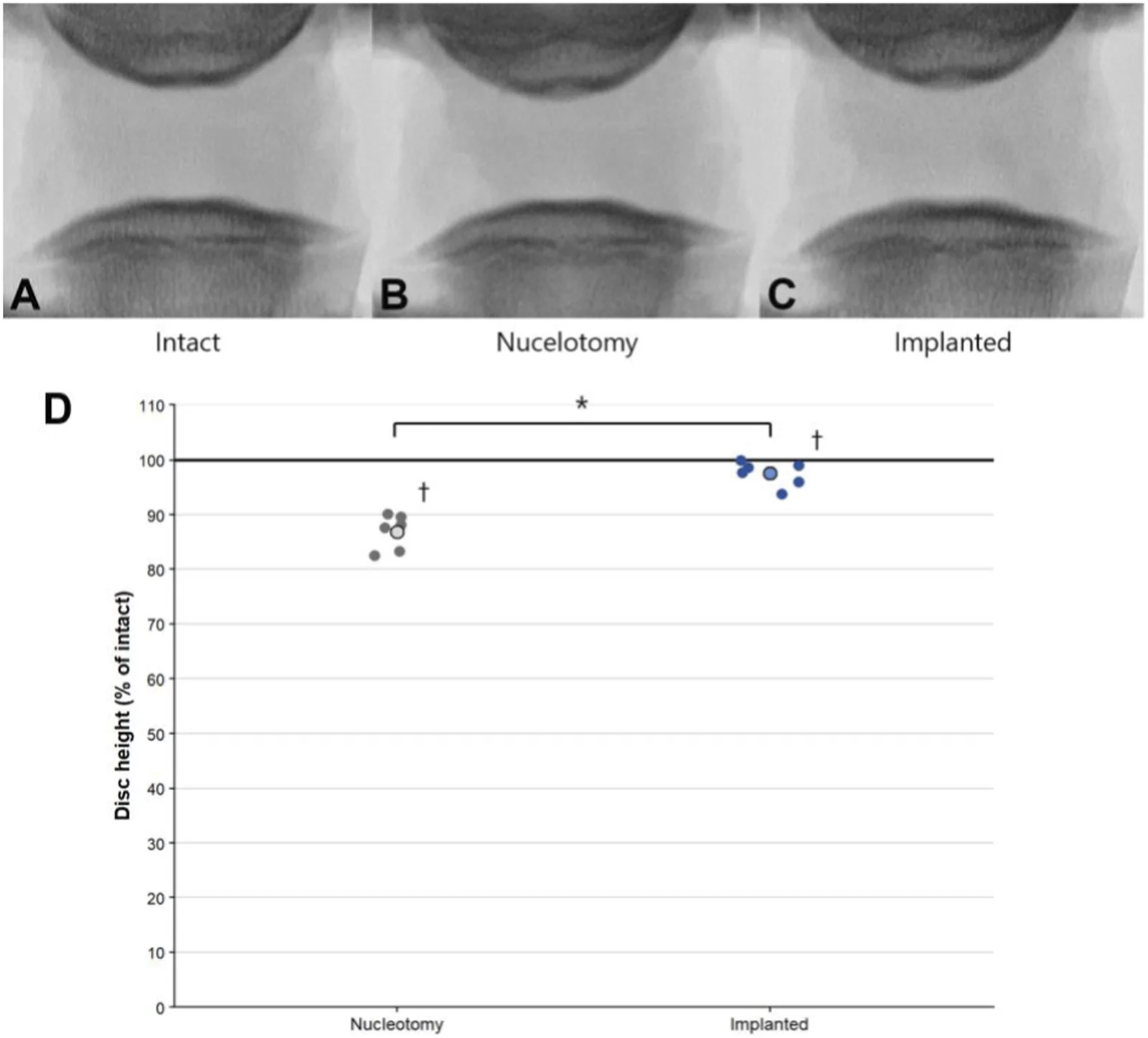
Representative X-ray images of **(A)** intact, **(B)** nucleotomy, and **(C)** implanted discs. **(D)** Percentage changes in disc height relative to the intact condition for nucleotomy and implanted discs. Individual data points from independent experiments are shown as raw values, and group means are indicated as outlined circles. Experiments were performed in six independent biological replicates. *Statistically significant difference (*P* < 0.05). †Statistically significant difference compared with the intact condition (*P* < 0.05).

Representative bovine tail motion segment image and load–displacement curves for each experimental condition are shown in Figure 7. The ROM was significantly greater in the nucleotomy condition than in the implanted condition. In contrast, NZ stiffness was significantly lower, whereas NZ length was significantly greater in the nucleotomy condition compared with the implanted condition. Compressive stiffness and tensile stiffness did not differ significantly among the experimental conditions. However, slow ramp stiffness increased after nucleotomy and was restored to a value comparable to that of the intact condition following hydrogel implantation (Figure 8). Intervertebral disc height decreased by approximately 13% following nucleotomy compared with the intact condition, whereas hydrogel implantation restored disc height to a level comparable to that of the intact condition (Figure 9).

### 3.10 Pilot *in vivo* implantation in porcine model

**FIGURE 10:**
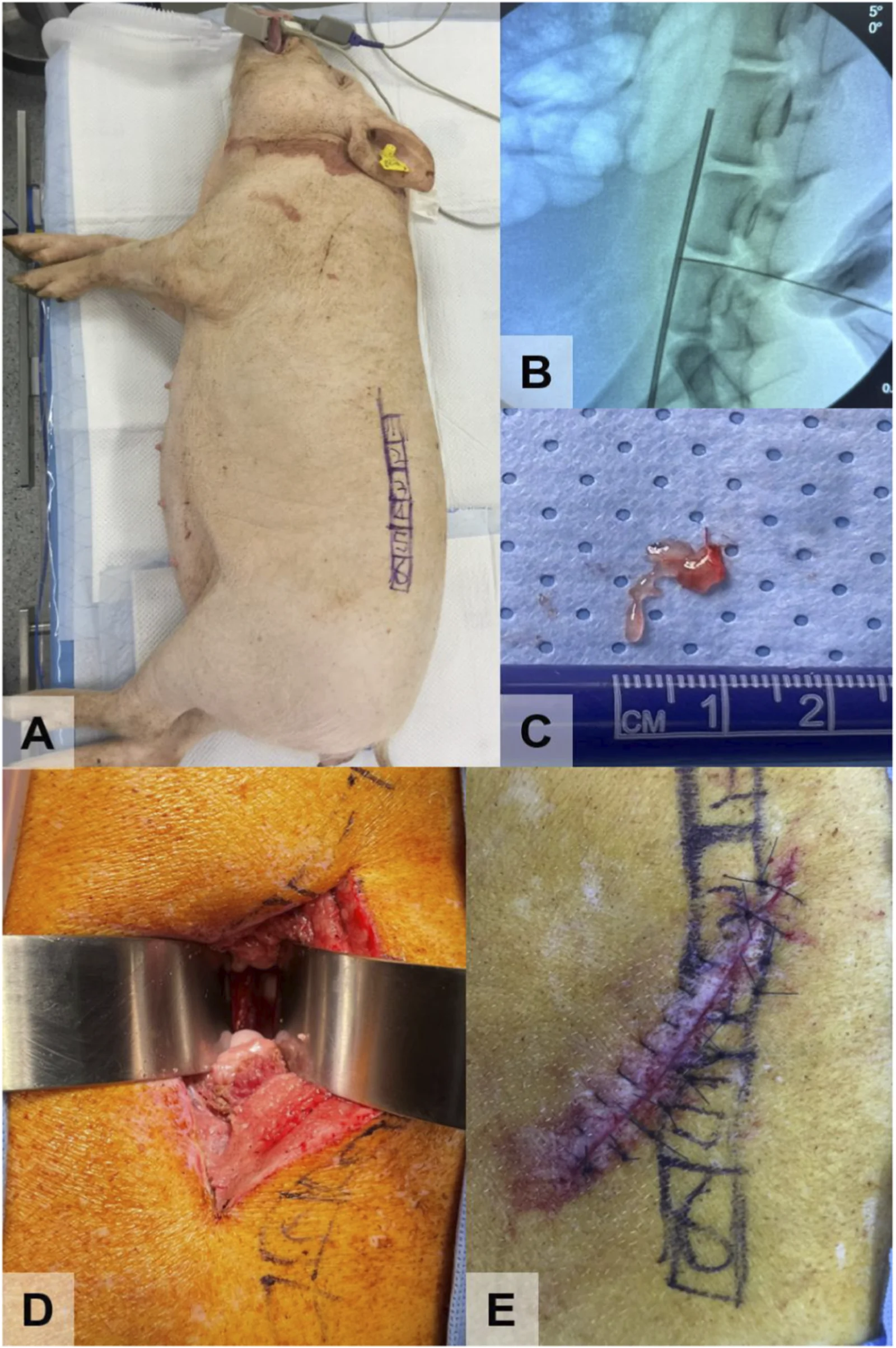
Representative images of pilot *in vivo* implantation of the tough hydrogel in a porcine model. **(A)** A pig with lateral position on the operating table. **(B)** Confirmation of the surgical level using C-arm fluoroscopy. **(C)** Resected clear, jelly-like normal nucleus pulposus of a porcine disc. **(D)** Deaver retractors were used to obtain a surgical field of view and approach to the disc. **(E)** Sutured skin with nylon. Three pigs were used in the experiment, and the L3–L4 and L4–L5 intervertebral discs of each animal were utilized, resulting in a total of six discs used for implantation.

During the surgical procedure, minimal intraprocedural bleeding was observed. The stitches were removed 2 weeks after surgery. During the 21-day observation period, no major complications, including nerve injury–related paraplegia, infection, or mortality, were observed. Representative surgical photographs are shown in Figure 10.

### 3.11 Pilot *in vivo* evaluation

**FIGURE 11:**
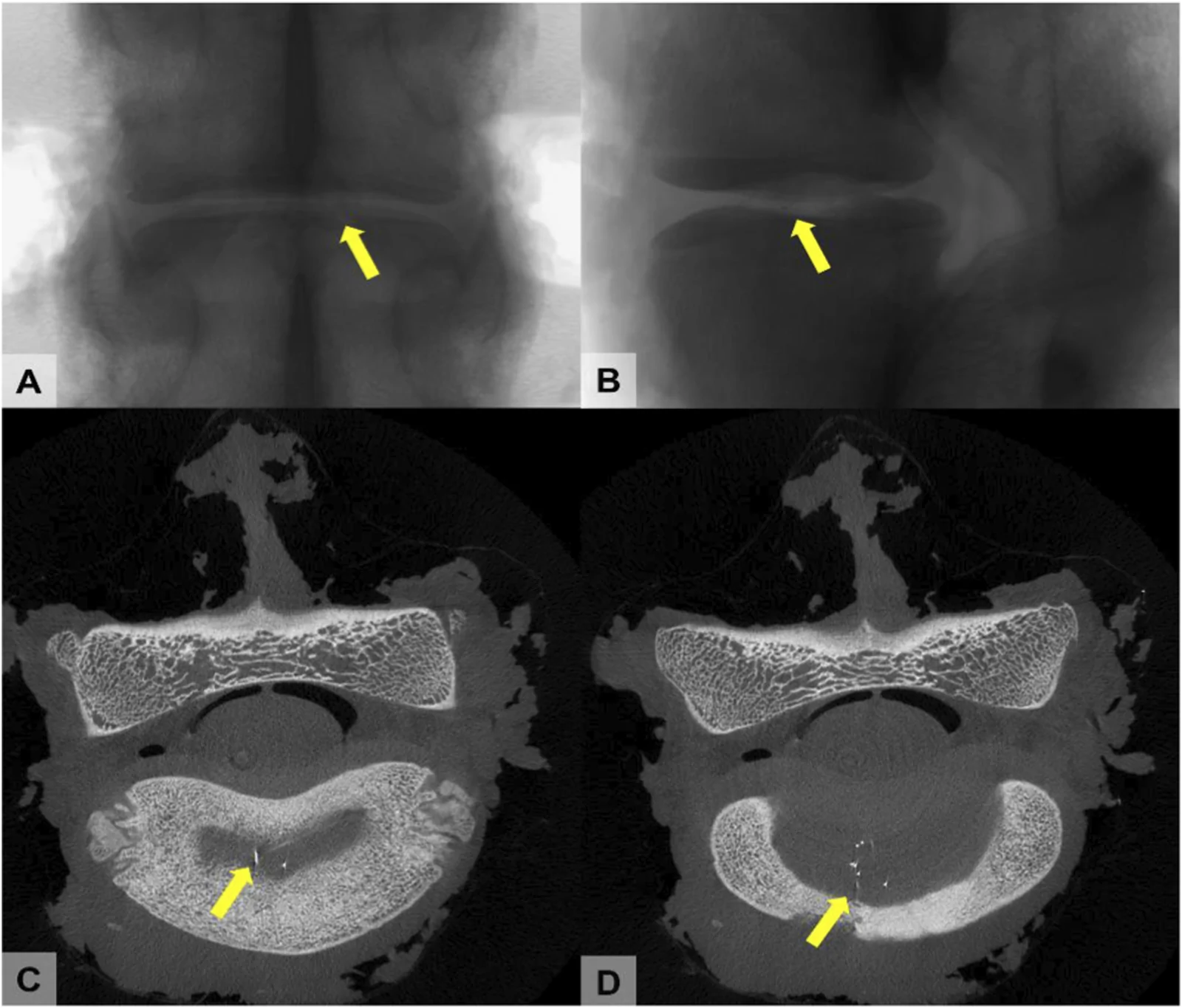
Representative X-ray and micro-CT images of the implanted tough hydrogel. A titanium fiber was detected in the central portion of the porcine L3-L4 disc using X-ray **(A,B)** and micro-CT **(C,D)** imaging. In one of the three pigs, a tough hydrogel containing a titanium fiber was used for X-ray and CT evaluation.

**FIGURE 12:**
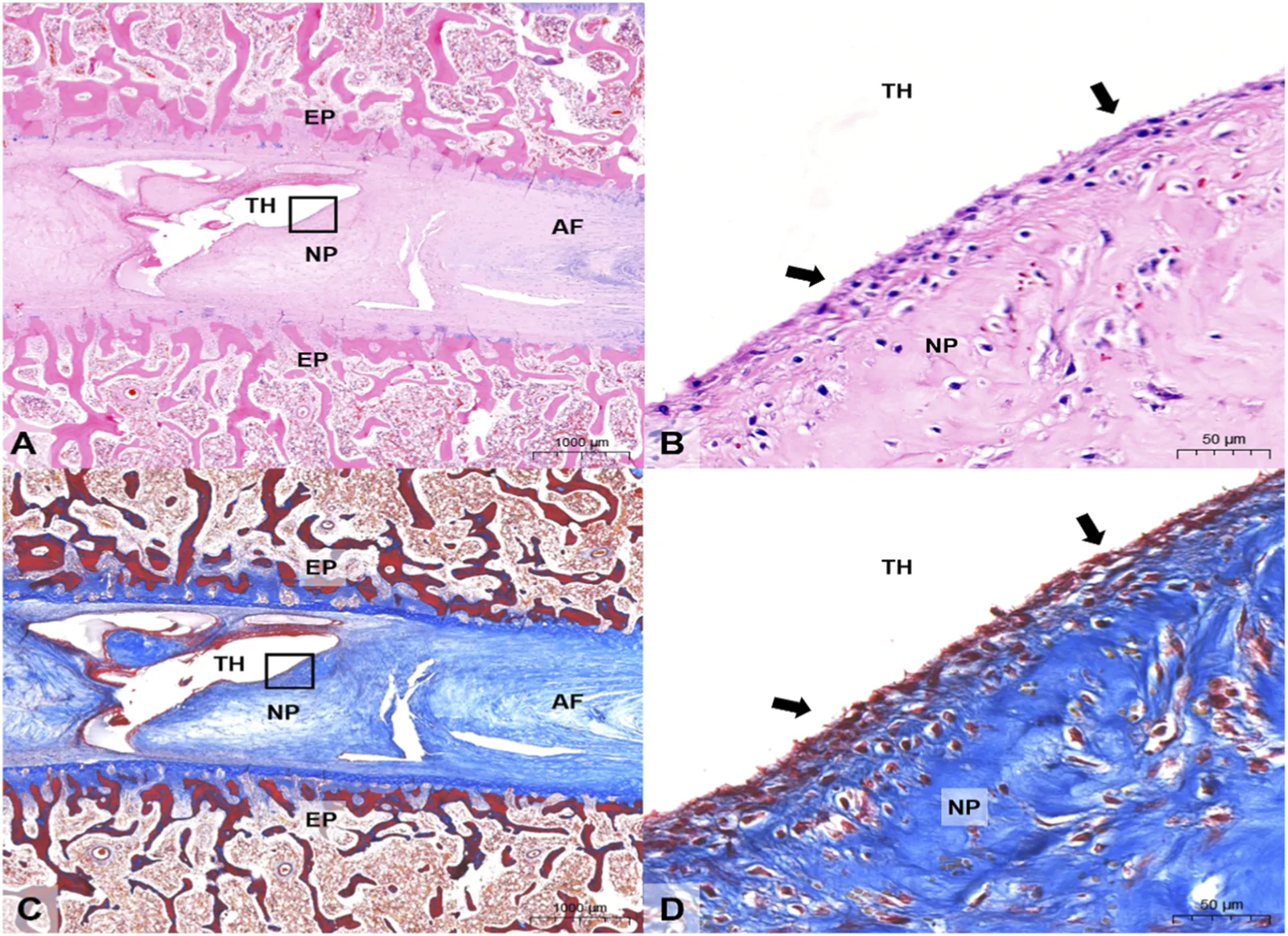
Representative histological images of the tough hydrogel 3 weeks after implantation in a porcine model. Hematoxylin and eosin stain for **(A,B)**, and Masson’s trichrome stain for **(C,D)**. The boxed areas in **(A,C)** (×10) are depicted at higher magnification in **(B,D)** (×170). Implanted hydrogel was surrounded by a thin fibrous capsule (black arrow) without apparent signs of abnormal immune reaction, showing close contact with highly collagenous remnant nucleus pulposus **(D)**. EP, endplate; NP, nucleus pulposus; TH, tough hydrogel; AF, annulus fibrosus.

X-ray and micro-CT evaluation of the two discs implanted with titanium fiber–reinforced hydrogel indicated no evidence of material migration outside the disc space (Figure 11). Six-disc *in vivo* histological evaluation indicated that the hydrogel was located within the nucleus pulposus, with preservation of the adjacent vertebral endplates and annulus fibrosus observed in all specimens. The implanted hydrogel was surrounded by a thin fibrous capsule, without apparent signs of abnormal inflammatory or immune reactions. It was found in close association with the highly collagenous remnant nucleus pulposus (Figure 12). Following implantation, the intervertebral discs exhibited moderate degenerative changes, with a mean Masuda score of 7.3, corresponding to mid-range degeneration according to the established grading system (Masuda et al., 2005).

## 4 Discussion

In this preliminary feasibility study, we conducted *in vitro* experiments, mechanical tests, and pilot *in vivo* implantation and evaluations to investigate the potential use of a reinforced tough hydrogel as a nucleus pulposus substitute. A mechanically reinforced tough hydrogel was developed using a PVA hydrogel and silk fibers, and *in vitro* experiments indicated an absence of acute cytotoxicity as well as support for basic cell adhesion and short-term proliferation. Mechanical testing showed that the tough hydrogel exhibited favorable swelling behavior, elastic properties, strength, and viscoelasticity. In a pilot large-animal study using pigs, the hydrogel remained within the disc during the short observation period without apparent signs of abnormal immune or inflammatory reactions.

Hydrogels are widely recognized for their favorable biocompatibility and are currently used in various products, such as contact lenses, wound dressings, drug delivery, and hygiene products (Caló and Khutoryanskiy, 2015). PVA hydrogels have minimal toxicity and are widely used in biomedical engineering research, particularly in applications, such as vascular stents and cartilage, owing to their strong and ultrapure characteristics (Bindu Nair, 1998; Adelnia et al., 2022). Silk also demonstrates high biocompatibility and is used as a medical material in various applications, including sutures (Jia et al., 2014). In this study, a cytotoxicity test was conducted on the final tough hydrogel product to evaluate potential risks. Even in the 100% tough hydrogel extract, quantitative cell viability exceeded 70% and the qualitative cytotoxicity grade remained below grade 2, meeting the standards set by ISO.

PVA/silk composite hydrogels have gained substantial attention in the field of tissue engineering owing to their favorable cell adhesion and proliferation properties (Kundu et al., 2012; Hou et al., 2020). In this study, silk fibers treated with 2-HEMA were utilized, and improved cell adhesion and proliferation were observed. These findings indicate the high biocompatibility of the tough hydrogel and its potential for application in tissue engineering, where the attachment of growth factors or bioactive molecules can regulate the proliferation and differentiation of the surrounding cells (Schmedlen et al., 2002). In addition, the PVA employed was a high molecular weight polymer matrix with an average molecular weight of 146,000 to 186,000 and a degree of hydrolysis of 99%, which was subjected to nine freeze–thaw cycles. Fully hydrolyzed PVA cryogels with high molecular weight are well-established as non-degradable materials (Chiellini et al., 2003; Wan et al., 2014), and consequently, the resulting robust hydrogel is anticipated to exhibit minimal degradation under physiological conditions.

Hydrogels are composed of hydrophilic polymers capable of retaining large amounts of water, resulting in their characteristic swelling behavior. The swelling property of hydrogels allows for easier and minimally invasive surgeries, as the hydrogel can be inserted into the body with fewer incisions when in a dry and compacted state. Reports have documented successful replacement after nucleotomy in porcine and canine model lumbar discs using dried-state hydrogels (Boelen et al., 2006; Bergknut et al., 2010). PVA hydrogels are known to develop a denser polymer network with repeated freezing/thawing cycles, resulting in reduced equilibrium swelling and water uptake while improving structural stability (Stauffer and Peppast, 1992). In our study, the hydrogel subjected to nine cycles reached sufficient swelling within 24 h and subsequently maintained a constant weight. The mean swelling ratio of the tough hydrogel was 202%, which was similar to the results mentioned in the aforementioned literature and was considered satisfactory at a desirable level. However, this evaluation was conducted under free-swelling conditions rather than physiologically relevant confined environments, and thus the results should be interpreted with caution.

Mechanical testing using bovine caudal motion segments, which closely resemble the human intervertebral disc, enabled evaluation of the biomechanical performance of the tough hydrogel implant within the annulus fibrosus-confined environment. Compared with material-level mechanical testing, this *ex vivo* model provides a more physiologically relevant assessment of the hydrogel as a nucleus pulposus replacement. Hydrogel implantation restored NZ stiffness, NZ length, and ROM to values closer to those of the intact condition than those observed after nucleotomy, indicating partial recovery of the biomechanical functions primarily governed by the nucleus pulposus. Likewise, the increased slow-ramp stiffness observed after nucleotomy was restored to a level comparable to that of the intact condition following hydrogel implantation, consistent with previous studies (Panjabi, 1992; Iatridis et al., 2013). In contrast, compressive and tensile stiffness showed no significant differences among the experimental conditions, likely because these parameters are largely governed by the annulus fibrosus under higher loading conditions (Johannessen et al., 2006). Overall, these findings suggest that the tough hydrogel can restore key biomechanical functions of the nucleus pulposus without adversely affecting the mechanical behavior of the intact motion segment.

The mechanical characteristics of the tough hydrogel provide additional context for interpreting the *ex vivo* biomechanical findings. The tough hydrogel exhibited a compressive elastic modulus of 3.75 kPa, which is within the range reported for human nucleus pulposus tissue (Umehara et al., 1996; Cloyd et al., 2007) and more than 10-fold greater than that of conventional PVA hydrogels (Pan et al., 2020). Because these values were obtained from unconfined compression testing, they should not be directly compared with biomechanical parameters obtained from confined compression or *ex vivo* motion-segment testing. Nevertheless, the results indicate that the hydrogel combines compliance with improved compressive load-bearing capacity compared with conventional PVA hydrogels.

In addition to compressive behavior, adequate tensile properties are important for both structural integrity and surgical handling. The tough hydrogel exhibited an ultimate tensile strength of 12.54 MPa and a tensile elastic modulus of 16.50 MPa, exceeding values reported for previously described PVA-based composite hydrogels (Hou et al., 2020; Xiang et al., 2022). Although the specific contribution of the 2-HEMA-treated core silk fiber was not independently evaluated, its incorporation may have contributed to the enhanced tensile properties. The resulting tensile stiffness also facilitated insertion of the fiber-shaped hydrogel through a small annular incision, suggesting practical advantages for minimally invasive implantation.

Previous nucleus pulposus replacement materials have shown a wide range of compressive properties but have achieved limited clinical success (Borges et al., 2011; Rasyida et al., 2025). For example, the Prosthetic Disc Nucleus (PDN) exhibited substantially higher compressive stiffness than native nucleus pulposus tissue and was associated with complications including endplate subsidence (Lindley et al., 2010; Wilke and Sciortino, 2025). Although the relationship between implant stiffness and endplate subsidence was not investigated in the present study, the compliant compressive behavior of the tough hydrogel may support more physiological load transfer. Further studies are needed to determine whether this characteristic influences long-term implant performance.

The native nucleus pulposus exhibits pronounced viscoelastic behavior (Iatridis et al., 1997; Leahy and Hukins, 2001; Izambert et al., 2003; Costi et al., 2008). Similarly, the tough hydrogel showed frequency-dependent increases in storage stiffness while maintaining relatively constant loss stiffness, resulting in tan δ values (0.09–0.15) comparable to those reported for native nucleus pulposus tissue (Gadd and Shepherd, 2011). In addition, the hydrogel exhibited minimal residual strain after prolonged cyclic loading, indicating favorable viscoelastic stability under repetitive mechanical loading.

Based on the aforementioned experimental results, we conducted a short-term large-animal pilot study to preliminarily evaluate the feasibility of the tough hydrogel as a nucleus pulposus substitute. Pigs are widely used in experimental and training settings in spinal surgery because their spinal anatomy closely resembles that of humans (Abbasi and Abbasi, 2019), and it has been reported that the loading applied to the lumbar spine of large quadrupeds such as pigs is not lower—and may even be greater—than that experienced by humans under vertical loading (Alini et al., 2008). Using this porcine model, we removed the lumbar disc nucleus pulposus and implanted the prepared tough hydrogel through an anterolateral approach, with no significant intraoperative complications observed. This observation is encouraging, as prior studies have reported that although hydrogels for nucleus pulposus augmentation can approximate native disc mechanical properties *ex vivo*, they frequently do not perform as intended when evaluated in large-animal models *in vivo* (Allen et al., 2004; Reitmaier et al., 2014; Detiger et al., 2016). Further studies are required to evaluate the long-term performance and translational potential of the tough hydrogel as a nucleus pulposus substitute.

Our short-term pilot *in vivo* evaluation indicated that the tough hydrogel remained well positioned within the nucleus pulposus region without a detectable inflammatory response during the observation period. The biocompatibility of both PVA hydrogels and silk has been well established, and our findings are consistent with previous reports (Gullbrand et al., 2017; Lin et al., 2019; Suryandaru et al., 2021). The present study provides additional evidence by employing a porcine model and performing a surgical procedure involving removal of the native nucleus pulposus followed by insertion of the tough hydrogel, rather than relying on simple hydrogel injection techniques. Another noteworthy finding is the absence of implant extrusion. Extrusion has been a major concern in nucleus pulposus replacement, as loss of implant containment not only reflects treatment failure but may also result in severe neurological complications—particularly if material migrates into the spinal canal, causing nerve compression and lower-extremity weakness (Wilke and Sciortino, 2025). The absence of extrusion observed in this short-term study suggests favorable initial containment of the hydrogel; however, the underlying factors contributing to this finding remain unclear. Therefore, these findings should be interpreted in the context of the limitations of this experimental model, and further long-term studies are warranted.

This study has several limitations. First, biological evaluation was limited to cytotoxicity, cell adhesion, and short-term proliferation assays, and therefore does not provide information on inflammatory response, extracellular matrix production, or nucleus pulposus cell phenotype, which should be addressed in future studies. Second, the intact annulus fibrosus may have been inadvertently damaged during hydrogel implantation. Even in degenerated discs with annular fissures, minimizing iatrogenic injuries is critical. Thus, future work should focus on methods that allow hydrogel insertion with minimal annular incision, potentially leveraging the swelling properties of hydrogels or alternative approaches. Third, postoperative antibiotics were administered for 7 days to reduce the risk of deep implant-related infection. Although a β-lactam antibiotic with minimal direct immunomodulatory effects was used, early inflammatory responses may have been partially masked. Additional limitations include the small sample size of three animals, the short 21-day observation period, the absence of a control group, and the lack of magnetic resonance imaging evaluation, which together limited a comprehensive assessment of long-term integration, durability, disc hydration, and implant positioning. Although there were no activity restrictions within the cage, housing the pigs in a larger enclosure would have more closely reflected human conditions and likely increased the reliability of the results. In addition, although the tough hydrogel is expected to be resistant to degradation due to its high molecular weight and complete hydrolysis, its *in vivo* degradation behavior and longevity have not yet been examined. Finally, the biomechanical behavior of the spiral-implanted tough hydrogel was not evaluated under confined intradiscal conditions. Although spiral-shaped nucleus replacement systems have previously progressed to clinical application (Husson et al., 2003), *ex vivo* confined compression testing comparing the hydrogel with the native nucleus pulposus was not performed in the present study and remains an important direction for future work.

## 5 Conclusions

In this preliminary feasibility study, the tough hydrogel created by combining PVA hydrogel and silk fibers was evaluated through *in vitro* assays, mechanical testing, and pilot *in vivo* studies. *In vitro* experiments demonstrated an absence of acute cytotoxic effects and showed that the material supports basic cell adhesion and short-term proliferation. Mechanical testing indicated favorable swelling behavior and promising elastic and viscoelastic properties. In the pilot large-animal model, the hydrogel could be surgically delivered and remained in place during the short observation period without evident acute adverse reactions. While these early findings indicate potential as a nucleus pulposus substitute, they remain preliminary. Additional studies evaluating inflammatory responses, extracellular matrix production, phenotypic maintenance, long-term biomechanical function, and extended *in vivo* outcomes will be required before its translational applicability can be fully established.

## Data Availability

The original contributions presented in the study are included in the article/Supplementary Material, further inquiries can be directed to the corresponding author.
