# Supplementary material for: A preliminary feasibility study of a tough hydrogel as a nucleus pulposus substitute for degenerative disc disease
Source: Front Bioeng Biotechnol. 2026 Aug 27;14:1821701. doi: 10.3389/fbioe.2026.1821701 (PMC13558221; doi:10.3389/fbioe.2026.1821701)
Supplement: Supplementary file 1 [file Supplementaryfile1.docx]

**Supplementary Table 1**. Qualitative morphological grading of cytotoxicity of extracts

| Grade | Reactivity | Conditions of all cultures |
| --- | --- | --- |
| 0 | None | Discrete intracytoplasmatic granules, no cell lysis, no reduction of cell growth. |
| 1 | Slight | Not more than 20 % of the cells are round, loosely attached and without intracytoplasmatic granules, or show changes in morphology; occasional lysed cells are present; only slight growth inhibition observable. |
| 2 | Mild | Not more than 50 % of the cells are round, devoid of intracytoplasmatic granules, no extensive cell lysis; not more than 50 % growth inhibition observable. |
| 3 | Moderate | Not more than 70 % of the cell layers contain rounded cells or are lysed; cell layers not completly destroyed, but more than 50 % growth inhibition observable. |
| 4 | Severe | Nearly complete or complete destruction of the cell layers. |

**Supplementary Table 2**. Masuda scoring system for disc degeneration based on histological analysis

| Findings | Score |
| --- | --- |
| I. Annulus fibrosus  Normal pattern of fibrocartilage lamellae (U-shaped in the posterior aspect and slightly convex in the anterior aspect) without ruptured fibers and without a serpentine appearance anywhere within the annulus  Ruptured or serpentine patterned fibers in less than 30% of the annulus  Ruptured or serpentine patterned fibers in more than 30% of the annulus | 1  2  3 |
| II. Border between annulus fibrosus and nucleus pulposus  Normal  Minimally interrupted  Moderate/severe interruption | 1  2  3 |
| III. Cellularity of the nucleus pulposus  Normal cellularity with large vacuoles in the gelatinous structure of the matrix  Slight decrease in the number of cells and fewer vacuoles  Moderate/severe decrease (> 50%) in the number of cells and no vacuoles | 1  2  3 |
| IV. Matrix of the nucleus pulposus  Normal gelatinous appearance  Slight condensation of the extracellular matrix  Moderate/severe condensation of the extracellular matrix | 1  2  3 |

**Supplementary Figure 1**. (A, B) Static compression test. (A) Before and (B) after application of compressive force. Flat jigs were used, and compressive force was applied at a speed of 2 mm/min. (C, D) Static tensile test. (C) Before and (D) after application of tensile force. Clamp-shaped jigs were used, and tensile force was applied at a speed of 5 mm/min.

**Supplementary Figure 2**. Cell cytotoxicity grade of (A) WI-38 and (B) disc cell for each material extract. Individual data points from independent experiments are shown as raw values, and group means are indicated as outlined circles. Each experiment was performed in three independent biological replicates.

*Statistically significant difference with P < 0.05.

PE, polyethylene; WI, Wistar Institute

**Supplementary Figure 3**. Results of the in vitro cell adhesion and proliferation test. (A) Absorbance of the CCK-8 reaction solution measured at 450 nm in the control and tough hydrogel groups after 4 h of incubation. (B) Difference in absorbance between the tough hydrogel and control groups (tough hydrogel minus control). Individual data points from independent experiments are shown as raw values, and group means are indicated as outlined circles. Each experiment was performed in three independent biological replicates.

**Supplementary Figure 4.** Dynamic mechanical analysis results of the tough hydrogel, showing (A) complex stiffness (K*), (B) storage stiffness (K′), (C) loss stiffness (K″), and (D) tan δ. Individual data points from independent experiments are shown as raw values, and group means are indicated as outlined circles. Each experiment was performed in three independent biological replicates.
